# Energy use and carbon footprints differ dramatically for diverse wastewater-derived carbonaceous substrates: An integrated exploration of biokinetics and life-cycle assessment

**DOI:** 10.1038/s41598-017-00245-1

**Published:** 2017-03-21

**Authors:** Yanbo Li, Xu Wang, David Butler, Junxin Liu, Jiuhui Qu

**Affiliations:** 1 Research Center for Eco-Environmental Sciences, the Chinese Academy of Sciences, Beijing, 100085 China; 2 University of Chinese Academy of Sciences, Beijing, 100049 China; 3 State Key Joint Laboratory of Environmental Simulation and Pollution Control, Research Center for Eco-Environmental Sciences, the Chinese Academy of Sciences, Beijing, 100085 China; 4 Centre for Water Systems, College of Engineering, Mathematics and Physical Sciences, University of Exeter, Exeter, EX4 4QF UK; 5 Key Laboratory of Drinking Water Science and Technology, Research Center for Eco-Environmental Sciences, the Chinese Academy of Sciences, Beijing, 100085 China

## Abstract

Energy neutrality and reduction of carbon emissions are significant challenges to the enhanced sustainability of wastewater treatment plants (WWTPs). Harvesting energy from wastewater carbonaceous substrates can offset energy demands and enable net power generation; yet, there is limited research about how carbonaceous substrates influence energy and carbon implications of WWTPs with integrated energy recovery at systems-level. Consequently, this research uses biokinetics modelling and life cycle assessment philology to explore this notion, by tracing and assessing the quantitative flows of energy embodied or captured, and by exploring the carbon footprint throughout an energy-intensive activated sludge process with integrated energy recovery facilities. The results indicate that energy use and carbon footprint per cubic meter of wastewater treated, varies markedly with the carbon substrate. Compared with systems driven with proteins, carbohydrates or other short-chain fatty acids, systems fed with acetic acid realized energy neutrality with maximal net gain of power from methane combustion (0.198 kWh) and incineration of residual biosolids (0.153 kWh); and also achieved a negative carbon footprint (72.6 g CO_2_). The findings from this work help us to better understand and develop new technical schemes for improving the energy efficiency of WWTPs by repurposing the stream of carbon substrates across systems.

## Introduction

Energy use and carbon footprints are acknowledged global-scale concerns associated with resource scarcity and climate change. Wastewater treatment plants (WWTPs) can cause an adverse effect because the aerobic removal of carbonaceous organic matters from the waste stream is energy intensive and is also highly related with airborne pollutants such as greenhouse gases^1–3^. As estimated recently, WWTPs in the United States consume ~15 GW annually^4^, and 50–70% of the electricity consumption is as a result of the aeration processes^5–7^, adding an unintended burden that increases energy use and carbon footprints. Yet, carbonaceous substrates could be converted to biomass, and ultimately utilized for power production^8, 9^. There is therefore a need to better understand the potentials of energy neutrality and carbon reduction in aerobic systems such as activated sludge processes (ASPs); and subsequently, to develop strategies to promote a sustainable reform in the role of WWTPs, from carbon-degradation oriented to carbon-recovery intensive infrastructures^10^.

Recently, the type of carbon substrate is recognized as one significant factor that affects the energy depletion and harvesting of traditional ASPs^11^. It was also observed in previous studies that the conversion of short-chain fatty acids (SCFAs) in the aeration processes was dominated by substrate storage and then polyhydroxyalkanoate (PHA) synthesis, rather than aerobic degradation to CO_2_, thereby promoting energy reduction in the water processing line^12^. Actually, the carbon sources present in wastewater cover a broad range of macro- and micro-molecules that include proteins, carbohydrates, and SCFAs, among others^13^. Moreover, sufficient concentrations of carbon substrates in ASPs are needed to enable microbial growth, and the metabolic pathways may vary among different microorganism; thus, this situation is complex. Consequently, little of the literature delivers extended information on the effect and metabolic characteristics of carbonaceous substrates, in relation to the energy and carbon footprints associated with ASPs.

Moreover, mathematical models have been widely applied to describe, predict, and evaluate the performance of an expanded range of wastewater treatment alternatives^14–17^. They provide a useful tool kit for gaining an in-depth understanding of the microbe-mediated processes and strongly support the design, implementation and optimization of the systems. Even so, little effort has been devoted to model and predict the role and behavior of carbon substrates within the context of energy and carbon implications in WWTPs.

Accordingly, this work aimed at providing fundamental information for further efforts on optimizing the current wide-applied technologies such ASPs, by altering and repurposing the influent stream of carbonaceous substrates at the system-levels, and eventually decreasing the expense of upgrading WWTPs with improved sustainability. Thus, it is not surprising that a biokinetics modeling approach was applied initially to describe the aerobically metabolic characteristics of several representative pure forms of carbonaceous source (i.e., proteins, carbohydrates, and common SCFAs) rather than a complex mixture of different substrates. Afterwards, the calibrated biokinetics data were used to simulate and forecast the transformation of the carbon substrates in a typical ASP. The detailed flows of energy consumed and recovered, and the carbon footprint of the entire treatment system with integrated energy recovery facilities, were then calculated, visualized and explained applying the philosophy of life cycle assessment (LCA). Overall, this work was intended to inform researchers, design practitioners, utility managers, and planners on how the carbonaceous substrate alters the energy use and carbon footprints of wastewater solutions; and on the associated implications for energy recovery practices.

## Results and Discussion

### Model Validation

**Figure 1:**
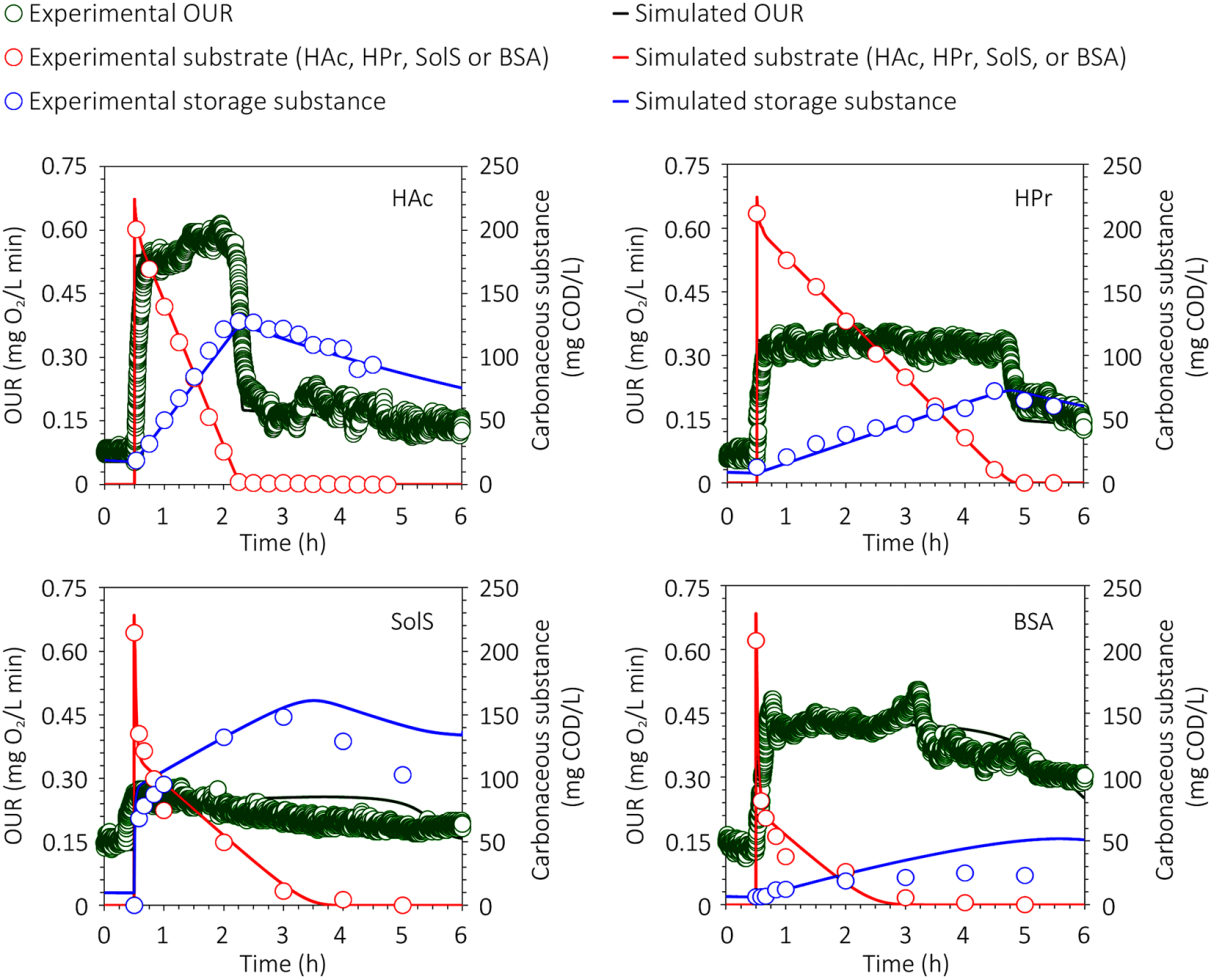
Model validation results using experimental data (influent carbon substrate, storage polymer, and OUR) from the four independent batch tests (measured data, symbols; model predictions, curves).

A sensitivity analysis was extensively applied to reduce the complexity of parameter estimation, determine the significance of model parameters, and pinpoint the dominant parameters^18^. As the parameters, i.e., substrate adsorption rate (*k*_*ads*_), half saturation constant for oxygen (*K*_*O*_), half saturation constant for readily biodegradable substrates (*K*_*S*_), endogenous respiration rate of active heterotrophic organisms (*b*_*H*_), endogenous respiration rate of storage substances (*b*_*STO*_) and fraction of inert particulate matter (*f*_*i*_) showed low variability in aerobic systems^19^, their values reported in the literature were directly adopted in this work. Figure 1 depicts the dynamic profiles of influent carbon substrate, storage product, and OUR in the ASPs during nearly 6 h of online monitoring, the data from which were used for the calibration of the other model parameters (see Table S1). Figure 1 shows a discrepancy between experimental data and model calibration in both the SolS- and BSA-driven systems, indicating a storage substance (*X*_*STO*_) deficit during the experiments. Though the model used herein to describe the production and consumption profiles of storage substance by starch and protein is relatively robust and rigorous amongst previously reported models, further research efforts are still needed to optimize the model and enhance the capability to trace complicated intermediates in the model. Thus, it is not surprising that a discrepancy existed between measured data and model predictions in the SolS- and BSA-driven systems of this work. Nevertheless, the model captures the parameter trends well, and the good fitting between measured and simulated data in Fig. 1 demonstrates the capability of the newly developed model in describing the microbial conversions of the studied substrates. Therefore, the verified model was used to perform processing modeling of all four ASPs, to acquire foreground data for further comprehensive analyses.

### Aerobic-Metabolic Behaviors of Different Carbon Substrates

Table 1 summarizes the aerobic evolution of carbonaceous substrates in the four ASPs. To ensure that all ASPs exhibited a similar substrate removal rate for better comparison, the performance of HAc-, HPr-, SolS-, and BSA-ASPs respectively operated at 1.6, 2.7, 4.4, and 6.8 days, was used for the subsequent comparative investigations.

**Table 1:** Aerobic evolution of carbon substrates among other substances in the four ASPs^a^.

Items	HAc-ASP	HPr-ASP	SolS-ASP	BSA-ASP
Influent carbon substrate (*S*_*S*_, mg COD/L)	200	200	200	200
Aerobic end carbon substrate (*S*_*S*_, mg COD/L)	2.0	2.3	5.9	5.5
Substrate metabolization by AHOs (mg COD/L d)^b^	2655.1	2810.2	3558.9	2980.9
Oxidation of *S*_*S*_ for AHOs growth (mg COD/L d)	446.9	729.4	406.6	694.4
Intracellular polymeric substance (IPS) synthesis (mg COD/L d)	1290.0	809.9	1527.0	776.9
Oxidation of *X*_*STO*_ for AHOs growth (mg COD/L d)	181.0	278.5	814.9	401.1
IPS accumulation (mg COD/L d)^c^	768.0	240.4	147.4	44.8
IPS accumulation/Substrate metabolization (−)	0.29	0.09	0.04	0.02
Aerobic end IPS (mg COD/L)	1253.0	655.2	647.6	326.9
Oxygen depletion for substrate metabolization (t O_2_/d)	7.4	9.9	8.1	11.1
Oxygen depletion for endogenous respiration (t O_2_/d)^d^	3.7	4.1	5.8	5.6
Heterotrophic CO_2_ generation (t CO_2_/d)	15.2	19.3	19.1	23.0

^a^Removal rate of initial carbon substrate greater than 97% in the ASPs was used as a benchmark for comparative evaluation; consequently, HAc-, HPr-, SolS-, and BSA-ASP were operated at SRTs of 1.6, 2.7, 4.4, and 6.8 days, respectively.

^b^Substrate metabolization includes the following three sub-processes mediated by AHOs: (i) oxidation of *S*_*S*_ for growth, (ii) utilization of *S*_*S*_ for IPS synthesis (*X*_*STO*_), and (iii) aerobic degradation of *X*_*STO*_ for growth.

^c^IPS accumulation rate equals to the difference of IPS synthesis, degradation, and self-respiration.

^d^Endogenous respiration herein includes the endogenous respiration of AHOs and self-respiration of *X*_*STO*_.

Obviously, SolS-ASP had a significantly higher substrate metabolization rate than BSA- and HPr-ASP (i.e., 3558.9 vs. 2980.9 and 2810.2 mg COD/L d, respectively), whereas HAc-ASP represented the lowest metabolization rate (2655.1 mg COD/L d). In this work, substrate metabolization includes three AHO-mediated bioprocesses (i.e., oxidation of carbon substrates for microbe growth, utilization of carbon substrates for intracellular polymeric substance (IPS) synthesis, and degradation of storage IPS for microbe growth). For the SolS-ASP, even though it illustrated the relatively highest IPS synthesis rate (1527.0 mg COD/L d) among the four ASPs, it also presented a greater IPS degradation rate for AHO growth than did BSA-, HPr-, and HAc-ASPs (814.9 vs. 401.1, 278.5, and 181 mg COD/L d). It was therefore not surprising that a much more powerful carbon substrate metabolism was observed in the SolS-ASP.

As further shown in Table 1, the IPS accumulation rate in HAc-ASP was much greater than that in the other three ASPs (768.0 mg COD/L d; over 3 times higher than that of the second greatest, HPr-ASP). The result illustrated that strong IPS synthesis exhibited in both the HAc-ASP and SolS-ASP, led to more carbonaceous substrate going toward the formation of storage products and facilitated IPS accumulation, even though IPS utilization and respiration also occurred simultaneously. The ratio of IPS accumulation to carbon substrate metabolization in BSA-, SolS-, HPr-, and HAc-ASP was 0.02, 0.04, 0.09, and 0.29, respectively (see Table 1). Apparently, the aerobic end IPS in the HAc-ASP was markedly higher than that in HPr-ASP, SolS-ASP and BSA-ASP (1253.0 vs. 655.2, 647.6 and 326.9, respectively). From this aspect, it was easily understandable that the less carbon substrate was degraded and utilized for microbial growth, the more carbon substrate was saved for intracellular substance transformation. This aligned with by far the lowest oxygen requirement (7.4 t CO_2_/d) and heterotrophic CO_2_ production (15.2 t CO_2_/d) present in HAc-ASP. This finding is consistent with results from our previous kinetic tests that indicated that a HAc-driven wastewater treatment system benefits from lower oxygen need and decreased CO_2_ production due to PHA synthesis^12^.

### Impact of Carbon Substrates on Embodied and Recovered Energy

Although this work only represents an initial step in determining the amount of energy flows within ASPs and expanded energy recovery systems, the findings confirmed that carbonaceous substrate and its existence forms would indeed significantly affect the energy implications of the investigated ASPs. For a typical ASP consisting of an aerobic bioreactor with recycling and a clarifier designed with nitrification and denitrification uninvolved, its capacity to remove nutrients had been shown to be significantly weak. Thus, it should be noted that the contribution of nutrient removal to embodied energy was neglected in the present work.

**Figure 2:**
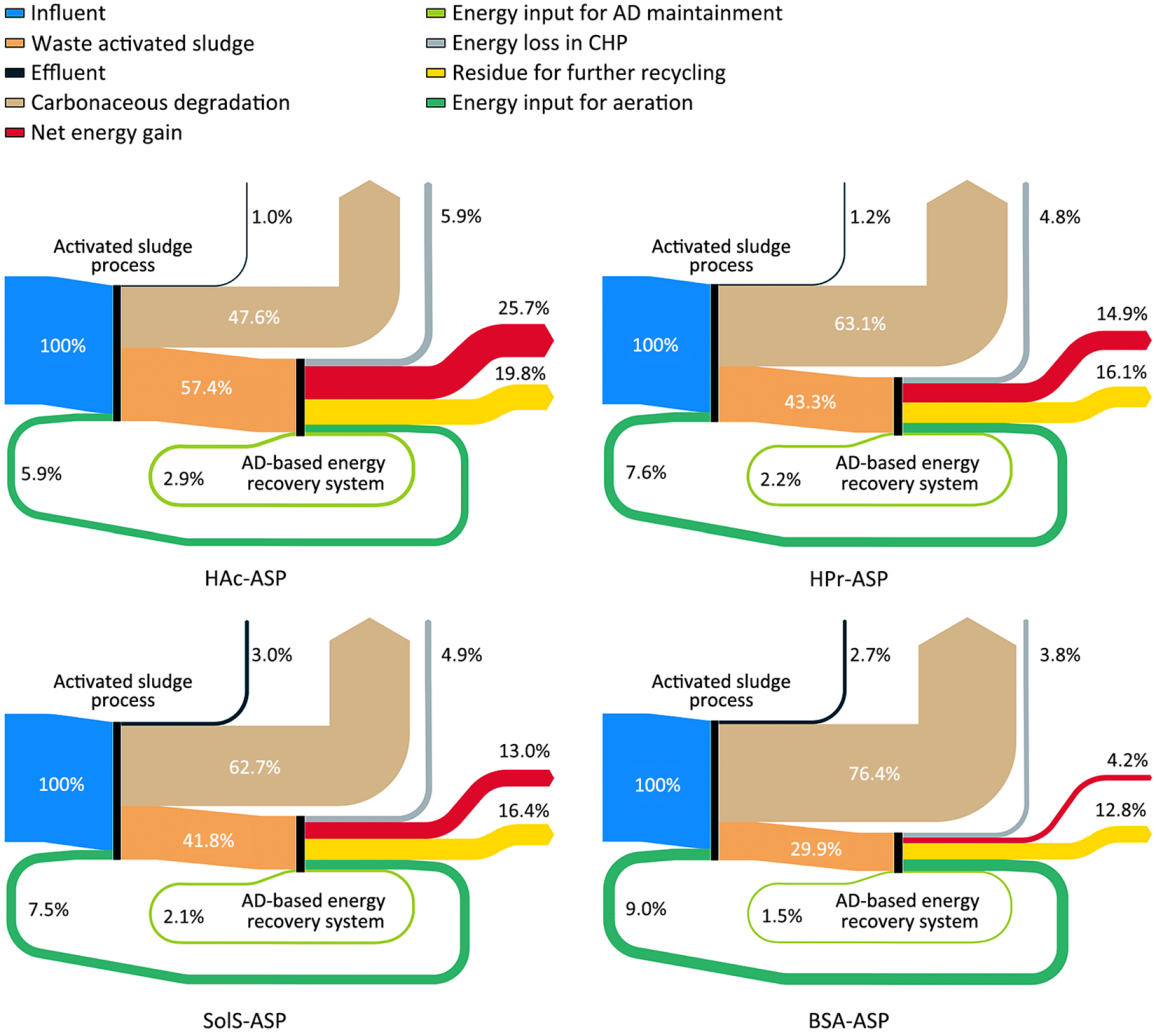
Quantitative visualized diagram tracing the embodied and recovered energy flow within each ASP system, through activated sludge treatment, and energy recovery and reuse processes, to the delivery of net energy gain for further end use. The energy potential of 0.772 kWh contained in the coming waste stream (200 g COD/m^3^) was used as benchmark (blue sky) for quantitative visualization.

As presented in Fig. 2, the embodied energy for system operation and maintenance increases from HAc-ASP (8.8%) to SolS-ASP (9.6%), HPr-ASP (9.8%), or BSA-ASP (10.5%), where over half was contributed by energy demand for aeration. Therefore, capturing more detailed information on energy and material usage for aeration system, operation conditions (e.g., oxygen transfer patterns, alternative blowers), and system performance would help in reducing the uncertainty of the embodied energy results. Carbon substrates contain chemical energy stored within their molecular bonds^12^, and the consumed energy associated with carbonaceous degradation in the aeration process thus increases with increased carbon degradation and utilization for microbial growth^20, 21^. Changes in the type of carbonaceous substrate have a larger impact on the unintended energy consumption associated with carbonaceous degradation than on the power demand for aeration. The energy potential consumed in the treatment stages of HAc-ASP and SolS-ASP was 0.367 kWh/m^3^ and 0.484 kWh/m^3^, respectively, whereas the energy lost in the HPr-ASP was 0.487 kWh/m^3^ and the BSA-ASP depleted 0.590 kWh/m^3^. Obviously, BSA-ASP had greater energy expenditure caused by carbonaceous degradation than did both HPr-ASP and SolS-ASP (76.4% vs. 63.1% and 62.7%, respectively); whereas HAc-ASP represented the lowest energy loss from aerobic degradation of a carbon source (47.6%). This result coincides with a formerly shown trend (see Table 1), whereby greater substrate utilization for microbial metabolism and significantly lower concentration of aerobic end storage substance, were both exhibited in BSA-ASP, while HAc-ASP represented the contrary.

**Figure 3:**
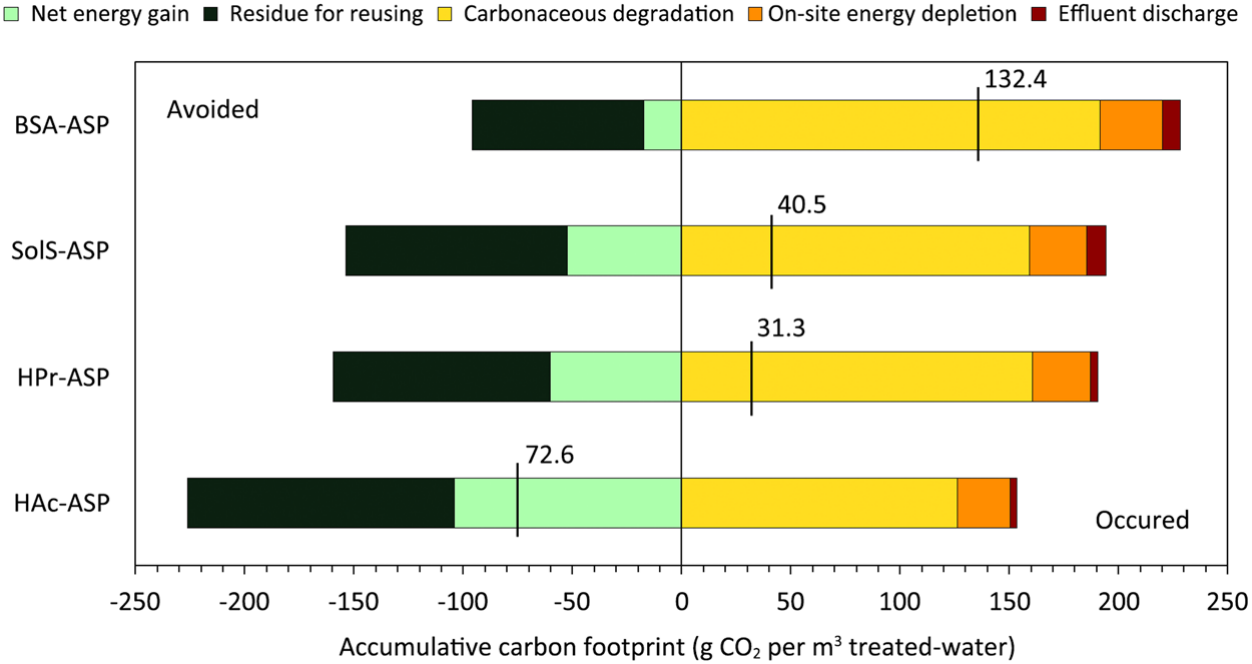
Accumulative carbon footprint for integrating wastewater treatment with expanded energy capture in all four ASPs. The green color series indicate the carbon emission avoided from bioenergy production, while the orange color series represent the carbon emission that occurred, including carbonaceous degradation in the ASPs, energy depletion for system maintenance, and also effluent discharge. Note that the black vertical segment presents the net carbon emission of the system boundary considered in each ASP.

Consequently, the chemical energy conserved and then transformed into the waste activated sludge (WAS) exhibited a greater level in HAc-ASP (57.4%) than in HPr-ASP, SolS-ASP, and BSA-ASP (43.3%, 41.8%, and 29.9%, respectively). This resulted in the greatest potential for further energy capture and recycling. Despite this, bioenergy harvesting from anaerobic digestion of WAS entirely satisfied the energy offsets for operation and maintenance of the systems in all four ASPs, reaching energy neutrality. Apart from the energy reused for operation of the aerobic and anaerobic systems, all the ASPs had net production of power from each system, whereas HAc-ASP and BSA-ASP, had respectively, the highest and lowest level (25.7% vs. 4.2%; or 0.198 kWh/m^3^ vs. 0.032 kWh/m^3^) among the ASPs explored. However, further research is still needed to determine how the offsets of embodied energy for varying energy recovery strategies change in relation to both scale and technologies. This is important because the scale of implementation has been observed to affect the energy consumed or recovered in wastewater treatment with integrated resource recovery^22, 23^. The residual biosolids released from the anaerobic digestion (AD) systems still have sufficient energy potential for further capture, ranging from 0.099 to 0.153 kWh/m^3^ (Fig. 3). This remaining potential will be discussed in the subsequent section on carbon accounting.

### Impact of Carbon Substrates on Carbon Footprint

The overall trend for the carbon footprint is similar to the total embodied energy because the direct emissions associated with carbonaceous substrate degradation in the treatment processes, and indirect emissions linked with electricity, are the dominant contributors (150.5–220.1 g CO_2_/m^3^; 150.5 g CO_2_/m^3^ for HAc-ASP, and 220.1 g CO_2_/m^3^ for BSA-ASP). These accounted for over 95% of the total carbon footprint (Fig. 3). It can be observed that the carbon footprint of the investigated systems fall into the range of the carbon footprint of WWTPs combined with resource recovery from a previous review of several case studies (0.1–2.4 kg CO_2_-eq/m^3^). However, this present work excluded the contributions of CH_4_ and N_2_O releases during treatment of wastewater; thus, the carbon credit of the BSA-ASP is still much lower than the top end of the range of emissions from historical studies^24^. This finding implies that reduced carbonaceous oxidation and consequently less energy intensive aeration (e.g., the HAc-ASP explored) could be beneficial for further improving energy efficiency. In activated sludge systems, CH_4_ contributions can be negligible due to the use of aerobic treatment processes for COD removal; yet, aeration requires additional electricity, highlighting an unavoidable trade-off between aerobic and anaerobic processes^25^. In this work, the CH_4_-contained biogas derived from WAS anaerobic digestion can also be overlooked because it was assumed to be collected and combusted to recover energy; nevertheless, it can also contribute significantly to the carbon footprint when emitted directly (0.40–0.95 kg CO_2_-eq/m^3^).

Incorporating energy recovery strategies was found to be beneficial for increasing the carbon footprint avoided through delivery of recovered energy products, such as heat and electrical energy. In Fig. 3, BSA-ASP had markedly lower potential for reducing carbon footprints by capturing bioenergy from WAS digestion than did HAc-ASP, HPr-ASP, and SolS-ASP (17.2 vs. 103.9, 60.1, and 52.5 g CO_2_/m^3^ avoided, respectively). This result aligned with the former outcome that BSA-ASP showed greater degradation of carbon substrates and only a little was saved and converted into WAS for further recovery. In contrast, BSA-ASP did not show an apparently lower capacity to mitigate carbon emission through harvesting energy from incineration of residual biosolids than did the other three ASPs. The main reason is that BSA-ASP had a relatively greater sum of endogenous residue content in its WAS owning to higher microbe metabolism in the systems (data not shown), which coincides with a previous report^12^. Overall, HAc-ASP was found to be the only alternative that could totally offset the carbon footprint caused by embodied energy through recovering energy from its WAS, achieving maximal level of net energy gain (0.351 kWh/m^3^), and yielding a negative carbon footprint (72.6 g CO_2_/m^3^).

### Prospects for the Future

The findings of this work highlight that the energy consumed or produced, and the carbon footprint caused, differs dramatically for varying wastewater-derived carbon substrates. The existing form and metabolic characteristics of the carbon source are further recognized as exhibiting a close relationship with embodied and captured energy in conventional wastewater treatment alternatives, such as the explored ASPs without nitrification and denitrification.

As estimated, many wastewater infrastructures at larger scale (such as city level) will need to be upgraded over the coming 10–15 years, and a new scheme incorporating reuse of wastewater-derived energy is recognized as a promising solution^26, 27^. Even if emerging technologies are able to potentially and simultaneously remove carbonaceous substrates and other waterborne substances, shift the energy balance, and empower net power production for other social-economic sectors^28, 29^. The economic costs and public acceptance, among other critical barriers, are great challenges that must be faced before this subversive reform is ultimately realized^29–31^. Thus, this work focused on discussion of another possibility that optimizes the current widely-applied technologies such as ASPs, by altering and repurposing the stream of carbon substrates at the systems-level, providing benefits for extending the service time of widely-accepted ASP technologies, reusing existing infrastructures, and potentially decreasing the expense of upgrading WWTPs.

This work highlights the capacity of acetic acid to facilitate maximal delivery of recovered energy products and to mitigate carbon accounting caused by the wastewater sector. Partial fermentation can occur in sewer systems, increasing the concentration of SCFAs^32, 33^, whereas primary settling affecting the particulate and dissolved carbon available for the subsequent carbon conversion^11^, these existing practices along with emerging systems provide numerious potentials to enable the implications of the present work. However, future planning could expand the life cycle framework to clarify how this method, if developed at different scales would influence cost, energy usage, carbon emissions, and other factors of concern for defined relationships. This extensive work will be much beneficial for future larger scale investigation. Further research is also needed to determine how the offsets of energy use and reduced carbon footprints for various wastewater-derived energy harvesting strategies, change with additional environmental impact categories. Moreover, this present work excluded the uncertainty accounting in the quantification of energy use and carbon footprint. It is understandable that this assumption would potentially contribute to the limitation of the work. For many parameters, not accurately knowing their underlying distribution functions, will hamper the smooth use of Monte Carlo simulation. Accordingly, future research efforts should also focus on elucidating sources of uncertainty and reducing uncertainty of the most sensitive parameters, in accounting both for energy use and carbon footprints, enabling robust life-cycle thinking, and determining to assist decision-making for wastewater management solutions with expected energy recovery practices.

## Methods

### Batch Experiments

#### Sludge and inoculum

For the present work, the sewage sludge was collected from a secondary clarifier of a WWTP in Northern China. The sludge characteristics are similar to those presented in our previous work^34^. The collected sludge was initially filtered using a 0.45 mm mesh to remove small particles, washed twice with distilled water, and finally stored at −4 °C for further respirometry tests. Four sequencing batch reactors (SBR) were started up with the collected activated sludge and were fed with the studied substrates i.e., HAc, HPr, SolS and BSA, respectively, for the enrichment of acclimated biomass for further utilizations. The experimental procedure was similar with a previous literature^35^.

#### Respirometry device

A schematic overview of the activated sludge respirometer is shown in Fig. S1 in Supplementary Information (SI), which was inspired mainly by previous literature^36^. Specifically, this device consisted of an open aeration vessel (4 L) and a magnetically stirred and sealed respiration chamber (2 L). During respirometry experiments, the activated sludge was continuously recirculated within the device at a flow rate of 0.75 L/min with a peristaltic pump (Longer® BT300-2J). Further, a heating system (COLE-PARMER® 12107-35) was used to maintain the temperature at 20 ± 0.5 °C in the aeration vessel, while a pH controller (EUTECH® alpha-pH800) was applied to sustain the pH at 7.5 ± 0.5 by the addition of 0.1 M HCl or 0.1 M NaOH. Dissolved oxygen (DO) determination can easily be disturbed by aeration or stirring in respirometry tests^37^. The online diagnosis and recalibration of DO probes are thus of significance in such tests. Accordingly, two additional measuring vessels (0.05 L each) were developed in the device and both equipped with a DO probe (WTW® CellOx 325). One was connected to the inlet and one to the outlet of the respiration chamber. A flow-switching unit consisting of solenoid valves and a time relay was designed, which allowed exchange of the contents of the two chambers. Finally, the signals of the DO probes were captured and logged on a computer equipped with the MultiLab software package.

#### Kinetic experiments

The acclimated SBR sludge (HAc-, HPr-, SolS- and BSA-fed) was added to four respirometer, respectively, at 1.0 g/L in 6 L and then aerated until the endogenous respiration phase was reached. At the same time, 20 mL of nutrient solution (g/L: NH_4_Cl, 32.0; MgSO_4_, 10.0; 6EDTA-2Na, 2.0; K_2_HPO_4_, 16.0; KH_2_PO_4_, 3.8; CaCl_2_·7H_2_O, 7.0) was added to the system, whereas 6 mL of allylthiourea (ATU, 30 g/L) was added to avoid growth limitation and nitrification based on historical literature^19^. Bovine serum albumin (BSA, Sigma-A7030), soluble starch (SolS, Sigma-S9765), sodium acetate (HAc, Sigma-S5636), and sodium propionate (HPr, Sigma-P1880) were used as substrate models for protein, carbohydrate, and common SCFAs, respectively, for the sake of mechanistic exploration. Four sets of the respirometry device were used and a substrate sample (BSA, SolS, HAc, and HPr) was added into the aeration vessel of each device at 200 mg chemical oxygen demand (COD)/L in 10 mL. The experiments were operated for approximately 6 h to trace and gain the data needed for further analysis. It should be further noted that the stability of the developed experimental system was also verified (*p* < 0.05) prior to the kinetic tests to ensure the system was robust and rigorous enough to support the whole experiments. The DO probes captured signals every 5 s and sent data to the PC system for quantifying the oxygen uptake rate (OUR) profiles according to Eq. 1^36^, whereas the two measuring vessels would be switched every hour for online diagnosis and recalibration of the probes, based on a previous approach^37^.

1 $${\rm{OUR}}=\frac{{\rm{Q}}}{{\rm{V}}}({{\rm{S}}}_{{\rm{O}},{\rm{in}}}-{{\rm{S}}}_{{\rm{O}},{\rm{out}}})-\frac{{{\rm{dS}}}_{{\rm{O}},{\rm{out}}}}{{\rm{dt}}}\,$$

where *S*_*O*,*in*_ and *S*_*O*,*out*_ were the oxygen concentrations entering and leaving the respiration chamber, respectively.

Samples (10 mL) of the mixture were taken at set intervals and then immediately treated with 6 M HCl to inhibit biodegradation. Subsequently, the treated mixture was centrifuged at 1000 r/min for nearly 15 min. Afterwards, the suppressant was filtered with 0.45 μm mesh for measurement of soluble matter (protein, carbohydrate, or SCFAs), while the centrifuged sediment was sampled to measure the glycogen and PHA contents.

### Analytic Methods

Measurements of pH, protein, carbohydrate, SCFAs, COD, and mixed liquid volatile suspended solid (MLVSS) content were performed as historically described^38^, whereas the glycogen and PHA contents were determined based on previous references^39, 40^. In addition, the conversion factor for BSA, SolS, HAc, HPr, PHA, and glycogen to COD was set as 1.40 g COD/g BSA, 1.12 g COD/g SolS, 1.07 g COD/g HAc, 1.51 g COD/g HPr, 1.67 g COD/g PHA, and 1.15 g COD/g glycogen, respectively^41–43^. Moreover, the results from this work are presented as the mean ± standard deviation of triplicate tests performed under the same conditions.

### Approach Package for Process Modelling

#### Model framework

For tracing and investigating the metabolic characteristics of the studied substrates, an integrated model framework was developed in the present work. The modelling of ASPs, particularly the biological substrate conversions, has evolved fundamentally in the last three decades from simple growth-based kinetics to more complicated models involving the description of storage phenomena^44^. To this end, a well-known simultaneous storage and growth (SSAG) model was used and calibrated subsequently for SCFAs^45^. However, since SSAG model was not develped to model and trace the conventional characteristics of both protens and strach, a specific model reported previously was selected and modified for the metabolism of proteins and starch^39^. Note that the nomenclature of all model components and parameters is the same as reported. The model used to describe the kinetic and stoichiometric behaviors of active heterotrophic organisms (AHOs) consists of several key processes: adsorption of slowly biodegradable substrate (*X*_*S*_, BSA or SolS); hydrolysis of adsorbed substrates (*X*_*Sads*_); storage of intracellular polymeric substances (*X*_*STO*_) on readily biodegradable substrates (*S*_*S*_, HAc, HPr, or other hydrolysis products); aerobic growth of AHOs (*X*_*AHO*_) on *S*_*S*_; aerobic growth of AHOs on storage products (*X*_*STO*_); endogenous respiration of *X*_*STO*_; and decay of AHOs. Note that the nutrients profile and its relevant mechanisms were not considered in the model. The stoichiometrics and kinetics of the modified model are summarized in SI (see Table S2).

#### Model calibration

The model includes seven specific biochemical processes and 27 stoichiometric and kinetic parameters, as given in Table S2. Before further numerical tests, the parameters were calibrated by employing a previously described systematic approach^46^. Specifically, the *X*_*AHO*_ concentration was first ascertained to provide an initial value for determining the endogenous OUR baseline and an endogenous respiration factor (*b*_*H*_, 0.2 d^−1^, ref. 47). Afterwards, the stoichiometric and kinetic parameters were estimated using the least square method, which minimizes the quadratic error between predicted and measured profiles of the substrates, storage products, and OUR profiles^48^. A version of AQUASIM 2.0 was used to determine the parameter surfaces^45^. In addition, parameter values were estimated by minimizing the sum of squares of the deviations between the measured data and the model predictions using the secant method embedded in the AQUASIM code. For each case, the model was first calibrated with one set of experimental data and then validated through simulation of the OUR for other sets of experimental data (not used for calibration) with the obtained best-fit parameter values with 95% confidence intervals.

#### Investigated systems

To mimic a representative ASP, an aerobic bioreactor with recycling and a secondary clarifier was modeled using our previous protocol^49^. ASPs were simulated and fed with the four different carbonaceous substrates (i.e., BSA-ASP, SolS-ASP, HAc-ASP, and HPr-ASP). Each ASP had the same influent COD level (200 mg COD/L). The hydraulic retention time of the aerobic reactor was fixed at 2 h, and the DO set point was 2.0 mg/L. Furthermore, the removal rate of initial carbon substrate greater than 97% in the ASPs was set as a benchmark for evaluation. All the simulations were run continuously in AQUASIM for over 2000 h to ensure the model components reached stable conditions (*p* < 0.05); next, the flow and composition details throughout the ASPs were acquired for further calculation.

### Calculations for Energy and Carbon Flows

#### Functional unit

As recommended by LCA literature and ISO 14040 guidelines^50^, a functional unit of 1 m^3^ of treated water was selected in this work, and treated water from all ASPs meet the same discharge requirement (i.e., the removal rate of influent carbon substrate >97%). The operation and maintenance phases for treatment and integrated energy recovery stages were mainly included in the system boundary. The power production avoided by energy recovery was considered via system expansion, in which coal for electricity production was considered an avoided product.

#### Energy flow model

A life cycle inventory of embodied and recovered energy was compiled for a comprehensive substance flow analysis of systems and key operating factors, which differ among the four ASP alternatives. The influent and effluent COD concentration of each ASP was employed to estimate the total energy contained in the influent and effluent stream by assuming a conversion factor of 3.86 kWh/kg COD^51^. The SSAG model was applied to determine the oxygen transfer rate (kg CO_2_/m^3^ of treated water), and the electricity consumed for aeration could be quantified by employing an aeration efficiency of 2 kg O_2_/kWh^12^. Energy demands to operate the sludge anaerobic digester included the requirements for heating. The amount of heat (kWh) required per 1000 kg of wet sludge was calculated from the difference between the assumed initial (20 °C) and desired (35 °C) temperatures, multiplied by the specific heat capacity of sludge with 6% solids (4.18 kJ/kg °C)^48^, and the heat loss from the AD system with available heat transfer coefficients^52^. The energy requirement for stirring in the AD system was excluded because it required much less than that for the heating of sludge or heat loss from the AD system^53^. Furthermore, it should be noted that the biogas mostly containing CH_4_ and CO_2_ generated from AS system will be considered as a whole for onsite recovery of energy (including heat and electricty). Consequently, the energy recovery (kWh/m^3^ of treated water) from biogas combustion was determined from the biogas yield (m^3^) multiplied by the heat value of biogas (23 MJ/m^3^)^54^, and the total energy conversion rate (35 and 50% for heat and electricity, respectively)^48^ in a combined heat and power (CHP) system. The biogas figure was gained from the modeled sludge yield (kg/m^3^ of treated water) multiplied by the modeled volatile solids (VS) and the biogas yield rate (0.65 m^3^/kg of VS)^54^.

#### Carbon Accounting

It should be noted that the carbon emission in this work was estimated from energy consumption, on-site and excess carbonaceous degradation, and carbon release avoided through harvesting of bioenergy from biogas combustion and incineration of residual biosolids. As the biogas derived from WAS anaerobic digestion was viewed as a whole for energy recovery, it can be overlooked in carbon footprint accounting; however, a CO_2_ emission factor of bioenergy conversion was still involved herein. Specifically, the CO_2_ production factors from coal-based energy production and bioenergy recovery were 877 and 353 g CO_2_/kWh, respectively^55^. The CO_2_ emission factor of sludge incineration was assumed to be 0.415 kg CO_2_/kg of sludge^56^. The on-site carbonaceous degradation and consequent CO_2_ release in ASPs was obtained from the SSAG model, whereas the contribution of excess carbonaceous degradation from effluent discharge to aquatic systems was determined by the effluent COD concentration and an assumed conversion factor of 1.5 kg CO_2_/kg COD^57^.

## Electronic supplementary material

Supplementary Information
