# Supplementary material for: Energy use and carbon footprints differ dramatically for diverse wastewater-derived carbonaceous substrates: An integrated exploration of biokinetics and life-cycle assessment
Source: Sci Rep. 2017 Mar 21;7:243. doi: 10.1038/s41598-017-00245-1 (PMC5428015; doi:10.1038/s41598-017-00245-1)
Supplement: Supplementary file 1 — Supplementary Information [file 41598_2017_245_MOESM1_ESM.pdf]

Supplementary Information

(6 pages, 1 figure, 2 tables)

Energy use and carbon footprints differ dramatically for diverse wastewater-derived carbonaceous substrates: An integrated exploration of biokinetics and life-cycle assessment

Yanbo Li[1, 2, †], Xu Wang[1, 3, *, †], David Butler[4], Junxin Liu[1, 2], Jiuhui Qu[1, 5]

[1] Research Center for Eco-Environmental Sciences, the Chinese Academy of Sciences, Beijing 100085, China

[2] University of Chinese Academy of Sciences, Beijing 100049, China

[3] State Key Joint Laboratory of Environmental Simulation and Pollution Control, Research Center for Eco-Environmental Sciences, the Chinese Academy of Sciences, Beijing 100085, China

[4] Centre for Water Systems, College of Engineering, Mathematics and Physical Sciences, University of Exeter, Exeter, EX4 4QF, United Kingdom

[5] Key Laboratory of Drinking Water Science and Technology, Research Center for Eco-Environmental Sciences, the Chinese Academy of Sciences, Beijing 100085, China

[*]To whom correspondence should be addressed. Phone/Fax.: +86 62911425. E-mail: xuwang@rcees.ac.cn (X. Wang)

[†]These authors contributed equally to this work.

Notation

X_S	Slowly biodegradable substrates
X_{Sads}	Adsorbed substrates
S_O	Dissolved oxygen
S_S	Readily biodegradable organic substrates
X_I	Inert particulate organic material
X_H	Active heterotrophic organisms
X_{STO}	Internal storage product of heterotrophic organisms
$Y_{H,S}$	Yield coefficient for direct growth
$Y_{H,STO}$	Yield coefficient for growth on stored products
Y_{STO}	Yield of stored products on S_S
k_{ads}	Substrate adsorption rate
q_m	Saturation constant for absorption
k_H	Hydrolysis rate constant
f_{STO}	Ratio of substrate utilized for storage
q_{max}	Maximum substrate uptake rate
$u_{H,STO}$	Maximum growth rate on stored products
K_O	Half saturation constant for S_O
K_S	Half saturation constant for substrate S_S
K_{STO}	Half saturation constant for storage substance
K_X	Half hydrolysis saturation constant
b_H	Endogenous respiration rate of heterotrophs
b_{STO}	Endogenous respiration rate of storage products
f_i	Fraction of inert particulate substance

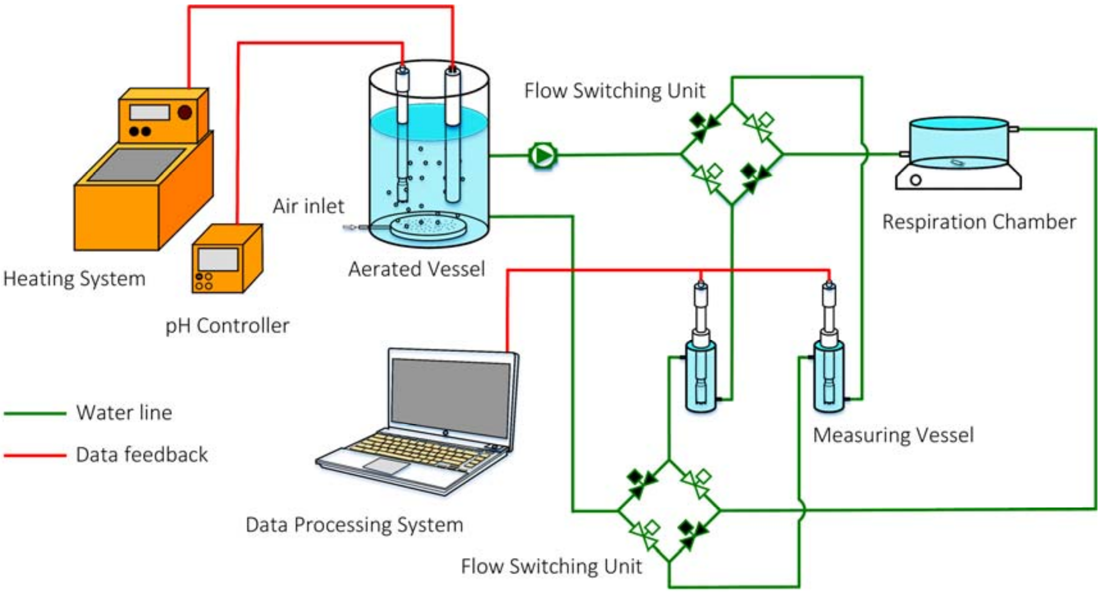

Figure S1 A schematic illustration of the proposed activated sludge respirometer.

Table S1 Kinetic and stoichiometric parameters for different substrates.

Parameters	Substrates				Source
	HAc	HPr	SolS	BSA	
$Y_{H,S}$ (mg COD/mg COD)	0.57	0.56	0.63	0.55	this study
$Y_{H,STO}$ (mg COD/mg COD)	0.65	0.63	0.65	0.60	this study
Y_{STO} (mg COD/mg COD)	0.81	0.76	0.92	0.74	this study
k_{ads} (h^{-1})	-	-	15.0	15.0	ref 1
q_m (mg COD/mg COD)	-	-	0.071	0.098	this study
k_H (d^{-1})	-	-	0.91	0.85	this study
f_{STO} (d^{-1})	0.67	0.45	0.72	0.45	this study
q_{max} (d^{-1})	4.4	1.8	-		this study
$\mu_{H,STO}$ (d^{-1})	1.1	1.3	1.2	1.1	this study
K_O (mg/L)	0.2	0.2	0.2	0.2	ref 2
K_S (mg COD/L)	0.7	0.7	-	-	ref 2
K_{STO} (mg COD/mg COD)	0.5	0.4	0.4	0.5	this study
K_X (mg COD/mg COD)	-	-	0.05	0.01	this study
b_H (d^{-1})	0.2	0.2	0.2	0.2	ref 3
b_{STO} (d^{-1})	0.2	0.2	0.2	0.2	ref 3
f_i (-)	0.2	0.2	0.2	0.2	ref 3

Table S2 Matrix of the proposed kinetic model.

Process	X_S	X_{Sads}	So	S_S	X_I	X_H	X_{STO}	Kinetics
Adsorption	-1	1						$k_{ads}\cdot\frac{q_m-(X_{sads}/X_H)}{q_m}\cdot X_S$
Hydrolysis		-1		1				$k_H\cdot\frac{(X_{sads}/X_H)}{K_X+(X_{sads}/X_H)}\cdot X_H$
Growth on S_S			$1-1/Y_{H,S}$	$-1/Y_{H,S}$		1		$f_{STO}\cdot q_{max}\cdot Y_{STO}M_O\cdot M_S\cdot X_H$
Storage of S_S			$1-1/\ Y_{STO}$	$-1/\ Y_{STO}$			1	$(1-f_{STO})\cdot q_{max}\cdot Y_{H,S}\cdot M_O\cdot M_S\cdot X_H$
Growth on X_{STO}			$1-1/Y_{H,STO}$			1	$-1/Y_{H,STO}$	$\mu_{H,STO}\cdot M_O\cdot\frac{(X_{STO}/X_H)}{K_{STO}+(X_{STO}/X_H)}\cdot\frac{K_S}{S_S+K_S}\cdot X_H$
Endogenous respiration			f_i-1		f_i	-1		$b_H\cdot M_O\cdot X_H$
Endogenous respiration of X_{STO}			-1				-1	$b_{STO}\cdot M_O\cdot X_{STO}$

M stands for a Monad kinetic function e.g. $M_S = S_S / (K_S+S_S)$.

References

1. Karahan, O.; van Loosdrecht, M. C.; Orhon, D. Modeling the utilization of starch by activated sludge for simultaneous substrate storage and microbial growth. *Biotechnol. bioeng.* **2006,** *94* (1), 43-53.

2. Sin, G.; Vanrolleghem, P. A. Extensions to modeling aerobic carbon degradation using combined respirometric-titrimetric measurements in view of activated sludge model calibration. *Water Res.* **2007,** *41* (15), 3345-3358.

3. Gujer, W.; Henze, M.; Mino, T.; van Loosdrecht, M. Activated Sludge Model No. 3. *Water Sci. Technol.* **1999,** *39* (1), 183-193.
